# The Effect of Peak Height Velocity on Strength and Power Development of Young Athletes: A Scoping Review

**DOI:** 10.3390/jfmk10020168

**Published:** 2025-05-10

**Authors:** Nikolaos-Orestis Retzepis, Alexandra Avloniti, Christos Kokkotis, Theodoros Stampoulis, Dimitrios Balampanos, Anastasia Gkachtsou, Panagiotis Aggelakis, Danai Kelaraki, Maria Protopapa, Dimitrios Pantazis, Maria Emmanouilidou, Nikolaos Zaras, Dimitrios Draganidis, Ilias Smilios, Antonis Kambas, Ioannis G. Fatouros, Maria Michalopoulou, Athanasios Chatzinikolaou

**Affiliations:** 1 Department of Physical Education and Sport Science, Democritus University of Thrace, 69100 Komotini, Greece; nretzepi@phyed.duth.gr (N.-O.R.); alavloni@phyed.duth.gr (A.A.); ckokkoti@affil.duth.gr (C.K.); tstampou@phyed.duth.gr (T.S.); dimibala10@phyed.duth.gr (D.B.); anasgkac1@phyed.duth.gr (A.G.); pangelak@phyed.duth.gr (P.A.); dkelarak@phyed.duth.gr (D.K.); mprotopa@phyed.duth.gr (M.P.); dpantazi@phyed.duth.gr (D.P.); maemmano@phyed.duth.gr (M.E.); nzaras@phyed.duth.gr (N.Z.); ismilios@phyed.duth.gr (I.S.); akampas@phyed.duth.gr (A.K.); michal@phyed.duth.gr (M.M.); 2 Department of Physical Education and Sport Science, University of Thessaly, 42100 Trikala, Greece; ddraganidis@uth.gr (D.D.); ifatouros@uth.gr (I.G.F.)

**Keywords:** maturation, talent identification, adolescent awkwardness, sports training

## Abstract

**Background**: Maturation is a complex biological process affecting all tissues, organs, and systems, particularly during adolescence. The Peak Height Velocity (PHV) period, a hallmark of adolescent growth spurts, is associated with individual differentiations in the development of performance attributes amongst youth. Understanding the influence of sports participation on strength and power during the PHV period is essential for optimizing training outcomes and reducing injury risk. This scoping review synthesizes the literature on the strength and power development in athletes during the PHV period across various sports, highlighting the interaction between maturation, training, and performance outcomes. **Methods**: A systematic search of PubMed and Scopus, supplemented by manual searches, identified peer-reviewed studies from 2004 to 2025. The included longitudinal studies involved structured training and assessed strength-related performance during and around the PHV period. PRISMA-ScR guidelines were followed. **Results**: Twelve studies met the inclusion criteria. It is found that strength and power are significantly affected during the PHV period and participation in sports mitigates these effects. Training characteristics such as training frequency/volume and sport specificity were key factors. Early maturers often outperformed late maturers, though adolescent awkwardness temporarily reduced performance around PHV. **Conclusions**: Systematic participation in sports training can limit the phenomenon of adolescent awkwardness in the performance of strength and power. Hence, training programs should be tailored to maturity status, emphasizing skill development, strength training, and injury prevention. Future research should explore individualized training and the mechanisms underlying performance variability during the PHV period.

## 1. Introduction

Maturation is a process that affects all tissues, organs, and systems of the human body throughout childhood and adolescence and physiological factors are in a continuous state of evolution [1,2]. This biological process is characterized by timing and tempo, resulting in a wide range of developmental differences, even among individuals of the same chronological age [3,4], indicating the different biological age of the youth. These differences become more pronounced when comparing athletes with contrasting maturity statuses, often defined as early or late maturers [5].

As age increases, the transition from childhood to adolescence and puberty brings significant changes to the human body, including alterations in shape, size, and composition—factors that are crucial for athletic success [6,7,8]. During the adolescent growth spurt, these changes occur for all individuals but vary in timing (chronological age at which age at Peak Height Velocity (PHV) occurs) [9], tempo (the rate at which maturation progresses) [9], and duration [5]. This rapid and intense transition begins between 11.6–12.1 years for girls and 13.8–14.1 years for boys, respectively. However, the average increment over the whole year around peak velocity, is considered as the PHV period [10]. During this period, growth in stature increases at a mean rate of 10 cm and a range of 6-13cm/year for boys and 8 cm and a range of 5–11 cm/year for girls, respectively [4,11]. PHV typically occurs between 9 to 15 years for girls and approximately 11.5 to 17 years for boys [2]. The estimation of age at PHV is a widely used measure of somatic growth, providing an accurate reference point for maximum growth rates during the adolescent growth spurt while also offering valuable information about other body dimension velocities [4]. The standard deviation for the mean chronological age at PHV is ±1 year, thus defining the population as average maturers (reaching PHV within one year of the mean population), as early maturers (reaching PHV more than a year before the population) and as late maturers (reaching PHV more than a year after the population) [11].

Not all body segments grow at the same rate during maturation. For instance, lower limbs grow faster than the trunk, a discrepancy that can significantly affect athletic performance [12], particularly interrupting the progress in performance attributes, where performance deterioration is observed in sprinting and jumping abilities [13,14]. This phenomenon is known to us as Adolescent Awkwardness and refers to a temporary delay or decline in motor control observed around the adolescent growth spurt or approximately six months before PHV [15,16,17,18]. This phase is characterized by changes in individual body part dimensions, impacting movement efficacy and efficiency. These alterations challenge an athlete’s ability to stabilize and control body segments and the forces required for movement. This leads to potential declines in coordination, static and dynamic control, and overall performance. Consequently, during the period of PHV, the risk of injury is increased compared to the pre- or post-PHV [15,16], where there is a dissociation between linear growth and bone mineral accrual, that marks a relatively long bone weakness) [19]. Simultaneously, around 6 months before and after PHV, reduced flexibility is observed in the lower limbs, which causes increased stress on the attachments of muscle tendons. These two factors ultimately lead to higher mechanical stress values in areas like tibial tuberosity [20] and thanks to the asynchronous growth patterns of body segments a disorientation of spatiotemporal landmarks is created [21].

Muscular strength is defined as an individual’s ability to produce maximum force, either statically or dynamically [12,22]. Strength development in boys and girls typically increases until approximately the age of 14. While girls often experience a plateau afterwards, boys undergo a strength spurt [12]. The timing and intensity of this spurt may vary among populations and specific strength components, but it is generally observed that peak strength velocity for most strength metrics begin 1.5 years before peak height velocity and peak 0.5–1.0 year after peak height velocity occurs [9], while also a peak point has been mentioned 2 years after PHV in boys [5,23,24]. Specifically, based on the linear percentage increase in strength during developmental stages, the maximum rate of strength increase is observed approximately one year after the year of PHV. Essentially, it appears that when children become accustomed to their body dimensions, they can apply greater amounts of force [9,25,26]. While this phenomenon is clear in terms of power, further investigation is required regarding strength through performance in strength exercises with greater demands on the neuromuscular system.

Muscle strength is closely linked to other performance attributes essential for successful sports participation, such as power, acceleration, speed, and change of direction (COD) [27,28,29]. However, it seems that periods of accelerated strength increases emerge during developmental ages, firstly during pre-puberty, where production of force relies more on the maturation of the neuromuscular system and appears to exhibit a linear percentage increase, which is expressed through more efficient intra- and intermuscular coordination. At a later stage, hormonal changes increase muscle mass and limb size, resulting in accelerated strength gains in the year of peak weight velocity [12,30,31]. Explosive strength for both upper and lower extremities follows a similar growth pattern, with peak development velocities occurring around PHV [23]. Speed and agility also peak during PHV, further underscoring the correlation between strength and other performance attributes [5].

Considering the variability and rapid changes during adolescence, especially around the PHV period, it is crucial to adapt talent identification criteria to account for current maturity status. Specifically, performance variation during the PHV period should be considered by talent identification experts, as transient changes in performance can occur. This approach ensures a fairer environment for less mature players within the same chronological age group, who might otherwise be disadvantaged by their early-maturing peers’ superior strength, power, and speed [18]. Fairer evaluation methods could allow late-maturing players greater opportunities to secure key roles or positions, reducing the likelihood of exclusion due to differences in physical development [32].

“Adolescent awkwardness”, combined with changes in joint stiffness, skeletal fragility, and potential imbalances, presents another significant challenge during PHV. These factors increase the risk of traumatic and overuse injuries during this period [33]. Therefore, accurately addressing the strength and performance development patterns during the maturation process, particularly during PHV, is essential. Such insights enable practitioners to design tailored and efficient training programs that meet the specific needs of athletes’ current maturity status. Nevertheless, the longitudinal development of strength is examined in the literature by evaluating performance in isometric or isokinetic strength. It is characterized by relatively simple motor patterns in their execution, such as grip strength and knee flexion-extension [34,35,36] and body weight exercises [37], while strength development in relation to performance in complex motor tasks remain unanswered. Additionally, the impact of strength training during developmental stages is well-documented, with percentage increases reaching or exceeding 300% over the course of one to two years of strength training [38,39]. However, there is a lack of focused information on athletes who participate in sports activities in contrast of those who are engaged in structured strength training interventions.

Previous systematic reviews have explored the relationship between maturation and performance attributes, particularly focusing on the effects of plyometric and resistance training in youth. For instance, Chen et al. [40] conducted a meta-analysis examining maturation-specific enhancements in lower extremity explosive strength following plyometric training in adolescent soccer players. Similarly, Ramirez-Campillo et al. [41]. investigated the effects of plyometric-jump training on performance attributes and sport-specific performance according to maturity levels. Additionally, Peitz et al. [42] systematically reviewed the impacts of resistance and plyometric training on performance attributes in youth, providing valuable insights from comparative studies. Although there is indirect information, strength performance is mentioned as a dependent variable in experimental designs, examining the impact of sports training on strength and power performance, particularly during the years of PHV [43,44,45]. The impact of various sports is noteworthy as the requirements in motor patterns are different, as well as the training load per sport (intensity, volume, etc.). Engaging in different sports may lead to different adaptations of strength during the PHV period.

Regarding power, which is a derivative of strength and velocity and is expressed through movements such as jumps, it has been found that the PHV period adversely affects children who are not engaged in sports [46]. Conversely, in soccer athletes, it has been established that this temporary decrease in performance is mitigated, attributed to training of the neuromuscular system. The studies emphasize the phenomenon of adolescent awkwardness and its partial mitigation through sports training [5,9]. While these studies offer important perspectives on how specific training modalities influence performance attributes across different maturation stages, they primarily focus on structured strength and plyometric training interventions. The significance of this study lies in addressing questions related to the development of strength and power during the sensitive PHV period, particularly concerning two attributes closely linked to the function of the neuromuscular system, which deteriorates during the PHV phase and contributes to adolescent awkwardness. Examining strength development in the years surrounding PHV may enable better design of training interventions during this specific maturation phase, as strength enhances sports performance by improving speed, agility, and jumping ability while also reducing the risk of musculoskeletal injuries. Therefore, the purpose of this scoping review was to explore the existing literature, critically appraise synthesizes the literature on the strength and power development in athletes during the PHV period across various sports.

## 2. Materials and Methods

This study did not require ethical approval. Following the guidance provided by the Joanna Briggs Institute, the study was registered on the Open Science Framework (OSF) on 14 February 2025 [47]. Furthermore, this scoping review was conducted following the 22-item guidelines of the Preferred Reporting Items for Systematic Reviews and Meta-Analyses extension for Scoping Reviews (PRISMA-ScR) [48]. The methodology ensured a systematic and comprehensive approach to gathering and synthesizing relevant studies.

### 2.1. Literature Search Strategy

The literature search combined structured searches in online databases with manual efforts to ensure thoroughness. The primary databases used were PubMed and Scopus, with additional records identified through manual searches of other sources.

For online searches, the terms included the following:

“Peak Height Velocity” OR PHV OR “Age at PHV” OR “circa PHV” AND Strength OR Jump* OR “stretch shortening cycle” OR power AND Young OR Youth OR Adolescent OR Child OR Children AND Players OR Athletes.

### 2.2. Eligibility Criteria

The review employed strict inclusion and exclusion criteria to ensure that only the most relevant and high-quality studies were considered.

#### 2.2.1. Inclusion Criteria

Only peer-reviewed journal articles in the English language were considered to ensure high-quality sources. The time frame for the literature review spanned from 1 January 2004 to 11 March 2025. The included longitudinal studies specifically examined the effect of maturation on strength in young athletes across various sports, focusing on how the PHV period influences performance attributes directly associated with muscular strength.

#### 2.2.2. Exclusion Criteria

To maintain focus and relevance, studies were excluded if they did not meet specific criteria. Non-English publications, conference proceedings, and studies lacking full-text availability were omitted to ensure accessibility and consistency in analysis. Additionally, studies with a duration of less than nine months were excluded to prioritize research that aligned closely with the review’s scope and objectives. Specifically, in most sports the duration of a single season occurs between September and May or June, consequently through a season long research design it can be feasible to evaluate the relevance of the changes of an entire training season [49,50]. In addition, most of the researches used the Mirwald equation for the estimation of PHV, which measures maturity offset within an error of ±1 year, 95% of the time and SD ± 0.5 years [4], allowing in that way to categorize individuals through the following three maturity stages; pre-PHV (<−0.5 years at PHV), circa-PHV (≥−0.5 years and ≤0.5 years at PHV) and post-PHV (>0.5 years at PHV) [51]. Therefore, a shorter experimental approach should be ineffective for achieving a different maturity status for the same athletes. Furthermore, reviews and meta-analyses were not included, as this scoping review aimed to synthesize original research rather than summarize existing secondary analyses.

### 2.3. Data Extraction

Two independent reviewers (N.R. and C.K.) conducted the data extraction process to ensure consistency and minimize bias. The workflow included the following steps:

- Duplicate Removal: All identified records were imported into Mendeley for deduplication.
- Title and Abstract Screening: Titles and abstracts were reviewed to identify studies that generally met the inclusion criteria.
- Full-Text Review: Shortlisted studies were examined in detail to determine their relevance based on specific criteria.

Any disagreements between the reviewers regarding study inclusion were resolved through discussion. Studies were included if they addressed specific parameters such as the type and duration of the study, the type of sport, the ethnicity, the chronological age, the baseline biological age of participants, the number of subjects, the test assessments used, the number of testing occasions, the key findings, and the training frequency/volume.

## 3. Results

**Figure 1:**
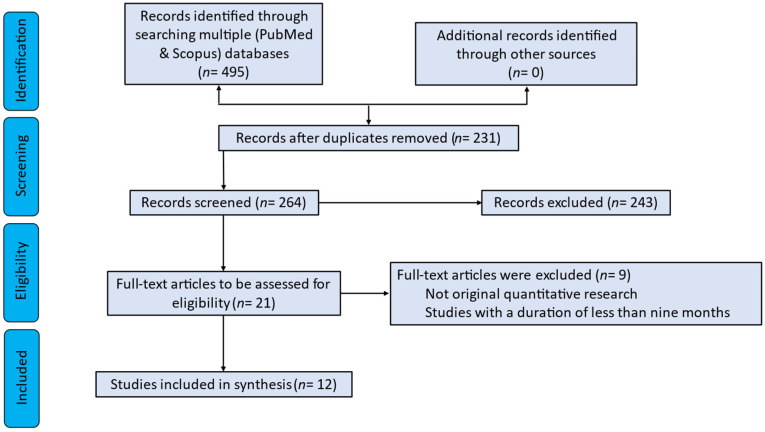
Workflow of screening methodology.

The search yielded 495 articles initially. After removing duplicates, 264 articles remained for title and abstract screening. Following a detailed full-text review, 12 articles were deemed eligible and included in the qualitative synthesis. A detailed workflow for the study selection process is illustrated in Figure 1, adhering to the PRISMA-ScR methodology to ensure transparency and reproducibility.

This scoping review synthesized findings from 12 studies that examined the effects of maturation on strength and power in young athletes across different sports. The results provide insights into how biological development impacts various physical attributes, particularly around the PHV period (Table 1). These findings were organized around four factors: the influence of training frequency/volume, sport specificity, biological age, individual variability in maturation.

Furthermore, the analysis of the included studies (Table 1) revealed a range of methodological approaches, participant demographics, and training conditions. Most studies employed mixed-longitudinal designs, with durations spanning from 9 months to 6 years. The participant’s age ranged from preadolescents (mean age 9.9 ± 0.4 years) to adolescents (mean age 14.0 ± 0.6 years). Baseline biological age assessments were consistent, with several studies referencing maturity offset (MO) and PHV periods. The assessments varied, focusing on strength (e.g., KE MVCiso, CMJ, HGS) and physical performance metrics. Across the studies, significant improvements were reported in key performance indicators, such as torque outputs, maximal velocities, and reactive strength indices. Training frequencies/volumes varied, with most athletes engaging in structured sessions ranging from 4.5 to 12 h per week. This variation highlights the diverse approaches to athletic development and conditioning across sports and age groups.

### 3.1. Improvements in Strength and Jump Performance Across Sports

Several studies highlighted significant improvements in muscular strength and jump performance during adolescence, particularly linked to maturation. Birat et al. [21] found that endurance-trained triathletes showed marked gains in knee extensor and flexor strength, as well as in muscle architecture metrics, over a 9-month training period. Similarly, Sekine et al. [52] reported increased jump height using the Abalakov test among basketball players, with high weekly training volumes emphasizing strength and sport-specific drills. For soccer players, Morris et al. [50] observed enhanced performance in countermovement jump (CMJ) and peak force during isometric mid-thigh pulls (IMTP) across different maturity stages, with training adapted to their level of biological development. Likewise, Bidaurrazaga-Letona et al. [49] found significant gains in CMJ height in youth soccer players over a 10-month period, demonstrating the influence of consistent training.

### 3.2. Maturation Timing and Performance Spurts

Maturation timing relative to PHV played a critical role in determining peak performance improvements. Guimaraes et al. [23] identified specific windows for peak velocity in various strength tests among basketball players. For example, sit-up performance peaked 6 months before PHV, while CMJ performance surged during PHV. This pattern underscores the importance of tailoring training interventions to capitalize on these critical developmental periods. Philippaerts et al. [5] similarly reported that physical performance metrics like trunk strength and vertical jump peaked during PHV while standing long jump performance peaked 18 months before PHV in soccer players. These findings highlight the dynamic nature of physical development and its relationship to maturation milestones.

### 3.3. Training Volume and Frequency as Key Performance Improvement Parameters

The role of training volume and frequency emerged as a significant factor in driving improvements. Ramos et al. [53] demonstrated that basketball players training approximately 6–7 h per week experienced consistent improvements in jump and throw performance. De Ste Croix et al. [54] also showed that reactive strength increased in soccer and basketball players who trained 6–12 h weekly alongside match play. In rugby, Till and Jones [55] found that vertical jump and medicine ball throw performance improved across different maturity stages, reinforcing the importance of consistent training during key developmental period. Similarly, Moran et al. [56] reported long-term gains in CMJ performance among elite soccer players, although training details were less explicitly described.

### 3.4. Longitudinal Trajectories and Developmental Trends

Long-term studies provided deeper insights into developmental trajectories. For example, Moran et al. [56] tracked English Premier League soccer players over six years, showing sustained improvements in jump performance, while training regimens were not consistently detailed. Till and Jones [55] highlighted significant longitudinal improvements in both jump and throw performance across a 5-year study, emphasizing the variability of developmental trajectories in rugby players.

**Table 1:** Employed studies.

Type of Study	Author	Year	Study Duration	Sport	Ethnicity	Chronological Age	Baseline Biological Age	Participants	Assessment(s)	Testing Occasions	Results	Training Frequency/volume
Longitudinal Study	Birat et al. [21]	2024	9 Months	Triathlon	France	(years) 14.0 ± 0.6 (CON)/13.9 ± 0.6 (END)	MO (years) −0.1 ± 0.7 (CON)/−0.1 ± 0.9 (END)	38 Males (23 Triathletes (END)/15 Archery (CON))	KE MVCiso/KF MVCiso/KE MVCcon/KF MVCecc	2	KE MVCiso Torque ↑/KE MVCcon Torque ↑/RF pennation angle ↑/RF muscle thickness ↑	At least 1 Swimming, 1 Cycling, 1 Running Session per Week (END)/<2 h (CON)
Mixed-Longitudinal Study	Ramos et al. [53].	2021	3 years	Basketball	Portugal	13.8 ± 0.4 U14/15.7 ± 0.4 U16	MO (years) 0.54 ± 0.7 U14/2.04 ± 0.6 U16	281 Males	CMJ/CMJ with Arm Swing/MBT/HGS	3	↑ in all variables	6.1 ± 1.6 h per Week (U14)/6.8 ± 2.5 h per Week (U16)
Mixed-Longitudinal Study	Guimaraes et al. [23].	2021	3 years	Basketball	Portugal	11–15	18 Months Before PHV/18 Months After PHV	160 Males	Sit-Ups/HGS/MBT/SJ/CMJ	6	Maximal Velocity: Sit-Ups 6 months before PHV/HGS, during PHV/MBT, during PHV/SJ 6 months after PHV/CMJ during PHV	4.5 to 6.0 h per Week
Mixed-Longitudinal Design	De Ste Croix et al. [54]	2021	3 years	Soccer and Basketball	United Kingdom	13.2 ± 0.5 U14/15.1 ± 0.6 U16	MO (years) −0.02 ± 0.68 U14/1.69 ± 0.71 U16	44 Males	5 maximum hop test	3	Reactive Strength Index ↑	5–7 Sessions per Week (6 to 12 h per Week) and 1 Match per Week (Soccer) or 2 Matches every two Weeks (Basketball)
Case Study-Longitudinal	Moran et al. [56]	2020	6 years	Soccer	United Kingdom	9.9 ± 0.4	MO (years) -3,1	6 Males	CMJ	18	Pre-Post CMJ ↑	Not Mentioned
Longitudinal Study	Sekine et al. [52]	2019	1 year	Basketball	Japan	13.1 ± 0.5 Late/12.8 ± 0.4 Mid/13.3 ± 0.6 Early	Estimated Age at PHV (years) (14.2 ± 0.6) Late/12.7 ± 0.4) Mid/11.9 ± 0.8) Early	41 Males	Abalakov jumps	2	Pre-Post ↑	12.5 to 14 h per Week/4.5 to 5 h S&C (Bodyweight Exercises, Sprint Training, Resistance Training, Stability Exercises)/8 to 9 h Sport-Specific Practice/1 to 2 Games Per Week
Mixed-Longitudinal Study	Bidaurrazaga-Letona et al. [49].	2019	10 Months	Soccer	Spain	12.3 ± 0.3 U13/14.0 ± 0.2 U15	MO (years) −3.12 ± 0.32, −3.18 ± 0.35 U13/−1.88 ± 0.45, −1.62 ± 0.80 U15	94 Males	CMJ with Arm Swing	2	Pre-Post ↑	3 Sessions per Week (1–1.5 h training per day)/1 Match per Week
Longitudinal Study	Morris et al. [50]	2018	9 Months	Soccer	United Kingdom	Elite 12.48 ± 0.7 Pre-PHV/14.24 ± 0.85 Circa-PHV/15.71 ± 1.16 Post-PHV/CON 11.57 ± 0.4 Pre-PHV/14.28 ± 1.29 Circa-PHV/15.83 ± 1.11 Post-PHV	MO/Elite −1.95 ± 0.63 Pre-PHV/−0.09 ± 0.64 Circa-PHV/1.52 ± 0.92 Post-PHV/CON −2.21 ± 0.62 Pre-PHV/0.17 ± 0.49 Circa PHV/2.13 ± 0.62 Post-PHV	150 (112 Soccer/38 Non-Elite Active)	IMTP/CMJ	2	CMJ ↑ among elite and control groups/Peak Force ↑ and Relative peak force ↑ in IMTP	4 Sessions and 1 to 2 S&C Sessions per Week (Pre, Circa-PHV)/6 Sessions and 2 to 3 S&C per Week (Post-PHV)
Mixed-Cross Sectional-Longitudinal Study	Till & Jones [55]	2015	5 years	Rugby	United Kingdom	12.8–15.5	MO (years) −2.5 to 2.5	121 Males	CMJ/MBT	1–4	VJ ↑ −2.5 and −1.5 and 0.5 and 1.5 Years from PHV/MBT	Not Mentioned
Longitudinal Study	Philippaerts et al. [5]	2006	5 years	Soccer	Belgium	10.4–13.7	MO (months) −24 to 24	33 Males	Sit Ups/BAH/SLJ/VJ	5	Maximal Velocity: Trunk Strength, during PHV/BAH, during PHV/SLJ, 18 months before PHV/VJ, during PHV	6 h Combined Competitive Play and Soccer Training per week (4 to 5 sessions) (Elite)/4 h (3 sessions per week) (Sub-Elite)/3 h (2 sessions per Week) per week (Non-Elite)
Longitudinal Study	Paschaleri et al. [57]	2024	18 Months	Track and Field	Greece	12.5 ± 0.29 Boys/10.5 ± 0.32 Girls	MO (months) −18	38 (20 Males/18 Females)	CMJ/EMG Medialis Gastrocnemius and Tibialis Anterior/Achilles Tendon Stiffness	3	Pre-Post: CMJ ↔/Tendon Stiffness ↑/Changes in EMG variables	Not Mentioned
Longitudinal Study	Radnor et al. [58]	2022	18 Months	Students (Rugby, Soccer)	United Kingdom	12.4 ± 0.2 Pre-PHV/13 ± 0.6 Pre-Post/14.3 ± 0.4 Post-Post	MO (years) −1.7 ± 0.3 Pre-PHV/−0.6 ± 0.4 0.7 ± 0.5 Pre-Post/0.8 ± 0.6 Post-Post	38 Males	CMJ	2	Larger changes: Post-PHV	Not Mentioned

Note: MO = maturity Offset; CON = control; END = endurance; KE MVCiso = knee extensor maximal voluntary contraction in isometric condition; KF MVCiso = knee flexor maximal voluntary contraction in isometric condition; KE MVCcon = knee extensor maximal voluntary contraction in concentric condition; KF MVCecc = knee flexor maximal voluntary contraction in eccentric condition; RF = rectus femoris; CMJ = countermovement jump; MBT = medicine ball throw; HGS = handgrip strength; PHV = peak height velocity; SJ = squat jump; S&C = strength and conditioning; IMTP = isometric mid-thigh pull; VJ = vertical jump; BAH = bent arm hang; SLJ = standing long jump; EMG; electromyography.

## 4. Discussion

This study aimed to examine sports participation as a parameter that affects the physiological development of strength and power during the PHV period, which is characterized by fluctuations in performance parameters and increased risk of injury. From the analysis of the literature, 12 studies emerged that met the criteria that were set out in the methodology. The analysis of the 12 longitudinal studies revealed consistent patterns regarding the physical development and performance outcomes of young athletes across various sports. A general increase in strength and power was observed across the studies, particularly during the phases surrounding PHV. The data analysis found that the level of maturity, sports participation, and training frequency/volume are factors that influence the development of strength and power during the period around the PHV.

The studies included in this review consistently examined the effects of maturation among athletes on strength over extended periods (nine months or more), enabling a comprehensive understanding of its influence in the years around the PHV period. Although the effect of strength training during developmental stages on physical performance attributes has been extensively studied [38,39,59,60,61,62] little is known about the specific influence of sports participation itself on strength and other performance outcomes. This review included studies where all participants were exposed to regular sports training and matches. It is known that in boys, muscle strength increases in a non-linear process, with the maximum rate of increase observed approximately 1.2 years after the PHV period and 0.8 years after the peak weight velocity [30]. When prepubescent athletes from various sports were compared with non-athletes of the same maturity status for handgrip strength and lower limb explosive performance, there were no significant differences between the groups. In this case, the lack of differences was possibly due to the similar anthropometric data, since the participants had not yet experienced rapid growth changes yet and secondly, training design at this stage is often characterized by moderate intensity and is focused on motor skills [63]. On the other hand, adolescents with consistent physical activity levels demonstrated higher strength performance than their sedentary counterparts. Remarkable is that data showed an average daily vigorous-to-moderate physical activity for non-athletes around 14 min, substantially lower than athletes’ training volumes [64]. The studies indicated that training frequencies/volumes, ranging from 4.5 to 12 h per week, were effective in enhancing athletic performance, while also highlighting variations based on sport type and athlete maturity levels. Similarly, judo athletes outperformed non-athletic individuals of the same maturity status for handgrip strength tests, indicating that the specificity of a sport can enhance the neuromuscular response and performance outcomes [65]. Soccer and basketball players demonstrated gradual improvements in reactive strength index from PHV to post-PHV, influenced by neuromuscular maturation and the athletes’ high training frequency (5–7 sessions and 1–2 matches per week) [54]. Therefore, it is revealed that neuromuscular function correlates directly with physical activity levels, and neuromuscular training close to or during the growth spurt period can positively affect strength improvements in non-athletic populations [66]. Moreover, Morris et al. [50] found that elite soccer players undergoing 4–6 weekly training sessions, supplemented with 1–3 strength and conditioning sessions/week, demonstrated superior strength gains compared to students engaging solely in physical education classes.

Despite the individual variability, it is clear that strength development in preadolescents depends on improvements in neural factors such as intramuscular and intermuscular coordination [31], and further increases that occur during adolescence are due to muscle hypertrophy [67]. Although maturity status is the primary contributor to strength performance, training should also be recognized as a significant influencing factor [68]. This positive effect of sports participation lies in the enhancements in the peripheral nervous system, indirectly contributing to greater strength values amongst trained and untrained individuals [45]. Considering the interaction of maturation and training, children and adolescents should maintain participation in structured sports training programs to become more efficient and effective.

Power, in the form of jumping ability and speed, is among the performance attributes most affected during the PHV period. In a study by Lloyd et al. [46], which involved 250 children aged 7 to 17 years, it was found that performance in tests related to the stretch-shortening cycle (jump height, reactive strength index, and leg stiffness) increased until age 11, decreased at age 12, and then increased from age 13 onwards. Additionally, the assessment of power development depends on the evaluation criterion, the part of the body being assessed, and the sport in which they participate. Lower limb explosive strength spurts varied by assessment type. Standing long jump velocities peaked at 18 months pre-PHV and around PHV, while CMJ performance consistently peaked during PHV period [5]. In studies comparing strength spurt timings, squat jump performance peaked six months post-PHV. In contrast, CMJ velocities peaked during PHV, likely reflecting the effects of plyometric drills and the repetitive jumping demands of basketball practice. Upper body strength gains peaked during PHV, possibly due to neuromuscular adaptations from repeated basketball-specific movements like passing, holding, and throwing. Improved coordination and synchronization of upper body segments also likely contributed to enhanced throwing ability [23]. The previous evidence was confirmed when athletes at the same maturity stages were examined using a drop jump evaluation. Results showed significant improvements in vertical jump height and maximum ground-reaction force from the pubertal to post-pubertal stage [69]. This confirms the hypothesis that sports participation and higher physical activity levels positively impact motor competency and musculoskeletal fitness [70,71]. It has been noted that athletes before and during puberty benefited from appropriate training, presenting ultimately superior strength, aerobic and anaerobic fitness, and speed compared to untrained individuals [12]. The importance of structured training during adolescence becomes more apparent when comparing athletes and non-athletes. Specifically, active adolescents consistently outperformed their sedentary peers in power-related measures, even though maturation itself remained the dominant factor influencing these differences, although CMJ performance which showed lower growth velocities during PHV than post-PHV. In a similar way, Paschaleri et al. [57] observed no changes in jumping performance from the pre-PHV period (−18 and −9 months from the PHV period) to the PHV period, despite the improvements in Achilles tendon stiffness and muscle activation during vertical jumps in track and field athletes. Radnor et al. [58] added to this understanding by showing that post-PHV athletes experienced greater CMJ performance improvements than their pre-PHV counterparts. This suggests that muscle architecture and maturation interact to enhance explosive power post-PHV.

The findings regarding sprinting ability and change of direction are likely due to the phenomenon of adolescent awkwardness during PHV, coupled with significant increases in fat-free mass during the adolescent growth spurt [53]. A longitudinal study, in which athletes were assessed before, during, and after PHV, revealed inter-individual variability in sprinting and jumping performance, that is related to PHV timing and tempo and severity of adolescent awkwardness [56]. However, training exposure appears to influence performance during puberty significantly [50], a period that is also associated with intense decreases in time to complete a certain small distance (12-14 years) [67]. Over a year, all PHV groups improved performance, with PHV basketball players demonstrating the greatest jump height gains. Morphological changes, including muscle growth, likely underpinned these strength improvements [52]. Strong correlations among weight, height, and strength between the ages of 10 to 14 reveal significant periods of development [72]. Radnor et al. [58] emphasized the role of morphological changes in enhancing explosive strength and sprint performance around and after PHV. Increased testosterone during adolescence also impacts strength and power-based activities, reinforcing the interplay of hormonal and physical development in athletic performance [70].

From the analysis of the literature, it is found that maturation affects the performance attributes of strength and power. It is characteristic that during the PHV period, there is a regression in the rate of improvement or even a decrease in performance, depending on the evaluation criterion. The modifying factors of this physiological development seem to be systematic involvement in sports, as it improves neuromuscular function and causes positive adaptations in the parameters of strength and power, which are also linked to motor patterns and the demands of sports. This can change with systematic involvement in sports as the neuromuscular system adapts to training requirements.

### 4.1. Limitations

This scoping review is subject to several limitations. Firstly, the studies included were limited to peer-reviewed articles published in English, potentially excluding valuable findings from non-English sources or gray literature. Additionally, while the review focused on athletes, the training programs and volumes were not consistently described across studies, making it challenging to conclude the specific effects of training regimens. Furthermore, individual variability in maturation, particularly among early and late maturers, was inconsistently addressed, which limits the generalizability of findings to diverse athletic populations. Moreover, the PHV method itself presents limitations, as its accuracy can be influenced by variations in biological maturity, especially among early and late-maturing adolescents [18]. This may affect the precision of timing assessments and the interpretation of physical development outcomes. Lastly, while the selected studies spanned various sports, certain disciplines were underrepresented, potentially narrowing the scope of this review’s conclusions.

### 4.2. Practical Implications

In brief, maturity status seems to be the most significant factor that affects strength development. However, training exposure through training volume and frequency, together with sport specificity, enhances the current strength level to a greater extent. Coaches and practitioners should consider the influence of PHV on strength development and related performance parameters when designing training regimens. Incorporating strategies to mitigate the effects of adolescent awkwardness, such as neuromuscular coordination exercises and strength-building programs, can help athletes navigate this critical developmental phase. Additionally, sport-specific practices should emphasize both upper and lower body strength development, considering the unique demands of each discipline. This approach can enhance performance while reducing the risk of injury during periods of rapid growth.

Moreover, the review highlights the role of consistent and structured training in optimizing strength and power gains. Coaches should monitor training frequency, volume and intensity to ensure adequate stimuli for development without overtraining, particularly during PHV. Programs designed with a balance of skill, strength, and conditioning components can maximize the benefits of this critical period.

### 4.3. Gaps and Future Research Directions

Several gaps in the current literature present opportunities for future research. Firstly, there is a need for more studies exploring the long-term effects of different training regimens on strength and performance across maturity stages, including comparisons between early, mid, and late maturers. Longitudinal studies with larger sample sizes and diverse athletic populations would provide a more comprehensive understanding of individual variability in strength development.

Future research should also investigate the underlying physiological mechanisms of adolescent awkwardness and how targeted interventions, such as neuromuscular training or plyometric exercises, can alleviate its impact on performance. Furthermore, studies examining non-athlete populations in greater depth, particularly regarding socioeconomic and environmental influences, could help identify barriers to optimal development and inform inclusive training strategies.

Lastly, interdisciplinary approaches incorporating biomechanical, hormonal, and psychological factors would offer a holistic perspective on the maturation process. Integrating data from advanced imaging techniques or machine learning models could provide deeper insights into how growth and training interact to shape athletic performance during adolescence.

## 5. Conclusions

This scoping review emphasizes the significant influence of maturation and the PHV period on performance attributes such as strength and jumping ability across various sports. Factors such as training frequency/volume, sport specificity, and biological age have a significant impact and directly affect the timing and magnitude of performance gains. Hence, it is important to monitor athletes’ performance at least during a single season (≥9 months), especially during the PHV period where body dimensions alter rapidly and the gradual increments of strength and power may be interrupted temporarily by the adolescent awkwardness phenomenon. Consequently, health practitioners and coaches should take into account the biological age of an individual while constructing proper training sessions, without over- or underestimating current performance capabilities of an athlete. Ultimately, encouraging children and adolescents to maintain a moderate to high weekly amount of sports training will help individuals perform better and utilize their potential.

## Data Availability

Not applicable.
